# Isolation and Partial Characterization of Macromolecular Urinary Aggregates Containing Carcinoembryonic Antigen-Like Activity

**DOI:** 10.1038/bjc.1974.93

**Published:** 1974-06

**Authors:** R. Nery, R. James, A. L. Barsoum, H. Bullman

## Abstract

**Images:**


Br. J. Cancer (1974) 29, 413

### ISOLATION AND PARTIAL CHARACTERIZATION OF MACROMOLECULAR URINARY AGGREGATES CONTAINING CARCINOEMBRYONIC ANTIGEN-LIKE ACTIVITY

R. NERY, R. JAMES, A. L. BARSOUM* AND H. BULLMAN

*From the Institute of Cancer Research: Royal Cancer Hospital, Chester Beatty Research Institute, Fulham Road, London, SW3 6JB*

Received 21 January 1974. Accepted 13 February 1974

**Summary.—Carcinoembryonic antigen-like substances in the urine of patients with bladder carcinoma and of healthy male subjects occur in a wide range of average molecular sizes. Some of these substances are glycoproteins giving reactions of antigenic identity with carcinoembryonic antigen (CEA) derived from colorectal carcinoma and occur in aggregation with proteins showing antigen specificities of albumin, haptoglobin and the heavy chains of immunoglobulins G, A and M. Recoveries of CEA-like activities, following Sepharose 4B chromatography of urinary materials of molecular size $> 3 \times 10^4$, varied from 40 to 1530%. Treatment with 1 mol/l $HClO_4$ caused an apparent solubilization of 85% of the CEA-like activity of such materials from the urine of a patient with bladder carcinoma and raised the specific CEA-like activity of the solubilized product to 379% of that of the untreated materials.**

THE oncofoetal relationship between an antigen first thought to be specific for carcinomata of the digestive system and foetal gut was first described by Gold and Freedman (1965). This carcinoembryonic antigen (CEA) or other antigenically related substances, were later shown to occur in comparatively low concentrations in a wide variety of normal tissues and in generally higher concentrations in tissues associated with increased cellular turnover as a result of injury or disease, especially in advanced and metastatic tumours and in a variety of non-neoplastic inflammatory or regenerative disorders (Martin and Martin, 1970; Freed and Taylor, 1972; Laurence *et al.*, 1972; Reynoso *et al.*, 1972; Hall *et al.*, 1972; Nery, Barsoum and Bullman, 1973). In particular, such substances have been detected by radioimmunoassay in the urines of patients bearing urothelial carcinomata (Hall *et al.*, 1972). Some of the properties of the urinary component having a mean molecular size (*i.e.* $2 \times 10^5$, Krupey, Gold and Freedman, 1968) similar to that of CEA derived from colorectal carcinomata have been described (Nery *et al.*, 1974). We now describe the isolation and some properties of macromolecular aggregates containing CEA-like activity from the urines of patients with bladder (urothelial) carcinoma and, in lesser amounts, of normal subjects.

### MATERIALS AND METHODS

*Chemicals and reagents.*—Sepharose gels and Blue Dextran 2000 were obtained from Pharmacia (GB) Ltd, London, W.5, gelatine-treated cellulose diacetate strips (Cellogel) from Reeve Angel Scientific Ltd, London, S.E.1, goat anti-human serum components from Miles-Seravac (Pty) Ltd, Maidenhead, Berks., acrylamide and NN′-methylenebisacrylamide from British Drug Houses, Poole, Dorset and rabbit IgG from Wellcome Research Laboratories, Beckenham, Kent. Other chemicals and reagents were from various commercial sources.

* Present address: Department of Medical Virology and Immunology, University of Essen, West Germany.

*Apparatus.*—Chromatographic columns equipped with cooling jackets were obtained from Pharmacia (G.B.) Ltd. The electrophoresis apparatus (Model U77) and analytical polyacrylamide gel-electrophoresis apparatus were from Shandon Scientific Co. Ltd, London, N.W.10 and ultrafiltration apparatus (Model 2000) and membranes were from Amicon Ltd, High Wycombe, Bucks.

*Distilled water.*—De-ionized double distilled water prepared in an all glass apparatus was used throughout.

*Aqueous buffer solutions.*—Phosphate buffered saline solutions contained $NaH_2PO_4$ (50 mmol), NaCl (150 mmol) and $NaN_3$ (3·1 mmol) per litre of solution adjusted to pH 5·8 or 7·2 with 1 mol/l-NaOH. Phosphate buffered saline-EDTA-rabbit IgG (pH 7·2) contained $NaH_2PO_4$ (22·5 mmol), $Na_2HPO_4$ (52·5 mmol), NaCl (75 mmol), EDTA (0·8 mmol) and rabbit IgG (1 g) per litre of solution. Barbitone (Veronal) buffer (pH 8·6) contained sodium barbitone (50 mmol) and barbitone (10 mmol) per litre of solution. Borate buffer (pH 8·6) contained $Na_2B_4O_7$, $10H_2O$ (25·5 mmol) and boric acid (75 mmol) per litre of solution. Borate buffered urea (pH 8·6) contained urea (5 mol) per litre of borate buffer.

*Urines.*—These were obtained from patients with urothelial carcinoma at the Royal Marsden Hospital. The specimens were collected in sterile containers and stored at −20°C until required.

*Goat anti-(carcinoembryonic antigen) antiserum.*—This, monospecific for carcinoembryonic antigen derived from colorectal carcinoma, was prepared from goats immunized with a standard purified preparation of the antigen as described by Darcy, Turberville and James (1973).

*Carcinoembryonic antigen.*—All preparations were from human colorectal carcinomata or their liver metastases. The standard preparation was a gift from Dr C. W. Todd (City of Hope National Medical Center, Duarte, California) and was labelled with $^{125}I$ as described by Egan *et al.* (1972). Other samples, kindly supplied by Dr C. Turberville (Chester Beatty Research Institute) were prepared as described by Krupey *et al.* (1968). The antigen is a glycoprotein (or a mixture of glycoproteins) of average molecular weight $2 \times 10^5$. Briefly, the antigen was prepared by extracting homogenized tumour tissue with aqueous 1 mol/l-$HClO_4$ and fractionating the solubilized components by successive gel filtration on columns (10 cm × 89 cm) of Sepharose 4B and Sephadex G200. Unless otherwise stated, carcinoembryonic antigen refers to preparations obtained in this way, without further purification. Generally, such materials show 60–100% of the antigenic activity in radioimmunoassay of the more highly purified standard substance.

*Radioimmunoassay.*—This was performed by the double antibody procedure of Egan *et al.* (1972) as modified by Laurence *et al.* (1972). Carcinoembryonic antigen-like activity of urinary preparations so determined is expressed as weight-equivalent antigenic activity, this being arbitrarily defined as that weight or concentration of the standard antigen required to give an equivalent inhibition in the radioimmunoassay. For brevity, this activity is referred to throughout the text as antigenic activity.

*Gel filtration chromatography.*—The packed columns were developed overnight, or longer, with phosphate buffered saline (pH 5·8) at 4°C until reproducible void volumes ($V_0$) (as determined by the exclusion of Blue Dextran 2000) were obtained. Fractions (25 ml) were collected from a Sepharose 4B column (bed dimensions 10 cm × 80 cm; $V_0$ 1·625 l) and fractions (4·7 ml) from a Sepharose 2B column (bed dimensions 2·5 cm × 56 cm; $V_0$ 140 ml) at a flow rate of 1·3–1·5 ml/h/cm$^2$ cross sectional area at 4°C. The $\epsilon_{-280}$ of effluent fractions was recorded continuously with an LKB Uvicord recorder or manually with a Unicam SP800 u.v. spectrophotometer. The antigenic activity of selected fractions was determined as a routine by radioimmunoassay. Appropriate fractions were combined, dialysed against several changes of distilled water at 4°C for 3 days and freeze dried.

*Disc electrophoresis on polyacrylamide gels.*—This was performed by the method of Ornstein and Davis (Davis, 1964). The sample (20–30 μg) was dissolved in the appropriate electrophoresis buffer (10–15 μl) containing, where appropriate, dissolved urea (50–75 μmol).

*Immunodiffusion in agarose.*—This was performed on glass microscope slides (7·6 cm × 2·5 cm) or in petri dishes coated to a depth of 2 mm with a 1·5% (w/v) solution of agarose in barbitone buffer. Preliminary immunotitration experiments were performed to establish optimum antigen/antiserum dilutions required for the formation of visible immunoprecipitates.

*Electrophoresis and immunoelectrophoresis in Cellogel.*—These were performed as described by Nery *et al.* (1974).

### RESULTS

### *Preliminary preparation of urinary fractions*

(a) Urines clarified by centrifugation at 2200 *g* at 4°C for 30 min were ultrafiltered through a PM-30 membrane in the Amicon ultrafiltration apparatus with stirring at 4°C. For each litre of urine, the material retained on the filter was washed with distilled water (2 × 100 ml) and dissolved in distilled water (25 ml). The resulting solution was dialysed in Visking tubing at 4°C for 24 h against one change of distilled water and freeze dried. This gave approximately 0·08–0·1 g and 0·85–1·1 g of freeze dried product/l of pooled urine (urine 6; Table) from healthy male subjects and of urine (urines 1–5, Table) from individual patients with bladder carcinoma respectively. The products showed 50 and 9–95 ng of antigenic activity/mg of residue, as determined by radioimmunoassay (Table, columns Sa of urine 6 and of urines 1–5, respectively).

(b) The aqueous solution (750 ml) of the material retained on the PM-30 membrane from a sample (30 litre) of urine 5 (Table) from a patient with bladder carcinoma contained 25·65 mg of antigenic activity as determined by radioimmunoassay. A sample (250 ml) of the solution was (i) dialysed and freeze dried as in (a) to give a product (9 g) showing 950 ng of antigenic activity/mg of residue. At 4°C, the remainder of the solution was (ii) treated with an equal volume of 2 mol/l-$HClO_4$, stirred for 30 min, centrifuged at 2200 *g* for 30 min and the clear supernatant dialysed in Visking tubing for 3 days against 6 changes of distilled water and freeze dried. The product (4·04 g) showed 3600 ng of antigenic activity/mg of residue. This indicated that the acid extraction procedure solubilized approximately 85% of the antigenic activity but increased the specific (w/w) antigenic activity of the solubilized product to 379% of that of the freeze dried product obtained from the same urine as described in (b) (i).

(c) Urine, collected by catheterization of the bladder of a female patient with bladder carcinoma, was treated with sodium azide to a final concentration of 0·02% (w/v) and divided into 2 equal portions. Corresponding portions were combined as collected and stored (i) at 4°C for approximately one week during collection or (ii) frozen at −20°C for a total period of approximately one month during, and after, collection. Sample (i) (6 l) was centrifuged at 2200 *g* at 4°C for 30 min and the supernatant solution concentrated to approximately 100 ml by ultrafiltration through a PM-30 membrane, diluted with distilled water (3 × 100 ml) and the volume of the diluted solution reduced to approximately 45 ml after each dilution step. The final solution was diluted with 0·1 mol/l phosphate buffered saline (pH 5·8, 50 ml), the total volume adjusted to 100 ml with distilled water and the mixture centrifuged as before. The supernatant solution, as determined by radioimmunoassay, contained 51 ng of antigenic activity/ml of solution, *i.e.* a total antigenic activity of 5·1 mg. Sample (ii) (6 l) was processed as in (a) above; the freeze dried product (5·5 g), as determined by radioimmunoassay, contained 950 ng of antigenic activity/mg of residue, *i.e.* a total antigenic activity of 5·2 mg or approximately 102% of the total antigenic activity recovered by procedure (c) (i). This indicated that freezing and thawing of the urine did not significantly alter the antigenic activity of the same urine not subjected to such processes.

### *Sepharose 4B chromatography*

A sample (4–6 g) of each freeze dried product obtained as described in (a), (b) and (c) (ii) above, dissolved in phosphate buffered saline (pH 5·8) at a concentration of 10% (w/v), was centrifuged on a bench centrifuge to remove small amounts of insoluble materials. Each resulting solution, the centrifuged final solution described in (c) (i) above and carcinoembryonic antigen (5 mg), obtained as a Sephadex G200 fraction from colorectal carcinoma (Krupey *et al.*, 1968) and dissolved in the same buffer (10 ml) were severally chromatographed on a column of Sepharose 4B. Representative elution profiles showing the main features of the results are shown in Fig. 1, as follows: (1) There was a wide distribution of antigenic activity, according to molecular size, in all the samples. (2) Two main peaks of antigenic activity (peaks A and C of curves 1, 2 and 3) were seen in all but one (see curve 4, Fig. 1, obtained from urine 4, Table) of the elution profiles: one (UCEA-3, peak A) was excluded by the gel and the other (UCEA-1, peak C) eluted almost coincidentally with CEA derived from colorectal carcinoma (curve 5). The single main peak of activity shown by urine 4 was of size intermediate between that of UCEA-1 and of UCEA-3. (3) Perchloric acid treatment of the fraction of urine 5 (urine 5a, Table) retained by the PM-30 membrane caused a relative decrease and increase respectively of the activities of peaks A and B (curve 3) in

FIG. 1.—Sepharose 4B chromatography of CEA-like urinary components: Elution of antigenic activity: curves 1 (○) and 4 (▽), urinary components of molecular weight approximately > 3 × $10^4$ from two patients with bladder carcinoma; curve 2 (△), similar components from healthy male subjects; curve 3 (●), a perchloric acid soluble fraction of similar components from a patient with bladder carcinoma; curve 5 (▲), CEA from colorectal carcinoma.

relation to those of the corresponding peaks (curve 1) of the untreated fraction of urine 5. (4) A specimen of urine obtained by catheterization of the bladder of a female patient with bladder carcinoma (see (c) (i) and (c) (ii) above), and hence uncontaminated with cervical secretions, showed an activity profile similar to that shown in curve 1. This profile was similar whether ((c) (ii) above) or not ((c) (i) above) freezing, thawing and freeze drying procedures preceded gel filtration. (5) Compared with the urines (urines 1–3 and 5, Table, curve 1) from the patients with bladder carcinomata, the pooled urine (urine 6, Table) from healthy male subjects contained relatively larger proportions of CEA-like components of mean molecular size smaller than that of CEA derived from colorectal carcinoma. The effluents from the Sepharose 4B column were combined into 4 fractions (A–D) as indicated in Fig. 1. Each combined fraction was dialysed at 4°C in Visking tubing against 6 changes of distilled water for 3 days and freeze dried. The antigenic activities of the freeze dried products and of the starting materials (Table) were determined by radioimmunoassay. The results (Table) showed a wide distribution of this activity in all the fractions, a wide variation (from 0·04 to 10·0) of the UCEA-3/UCEA-1 ($A_C/C_C$) ratio and recoveries of CEA-like activities ranging from 40 to 1530%. The highest relative proportion ($D_C/C_C$) of CEA-like components of molecular size smaller than that (*i.e.* $2 \times 10^5$, Krupey *et al.*, 1968) of CEA derived from colorectal carcinoma again occurred in the combined urines from healthy male subjects.

*Sepharose 2B chromatography*

A sample (10 mg) of UCEA-3 (17 mg), obtained after dialysis and freeze drying of combined effluents (fraction A, Fig. 1) of urine 5a (Table), was dissolved in phosphate buffered saline (pH 5·8; 5 ml) and chromatographed on a column of Sepharose 2B. Radioimmunoassay of effluent fractions showed (Fig. 2) 2 peaks of CEA-like activity: a major broad peak and a

FIG. 2.—Sepharose 2B chromatography: Elution of antigenic activity (○) and $\varepsilon_{280}$ (—) from the perchloric acid soluble Sepharose 4B sub-fraction A (Fig. 1) of urine (5a, Table) from a patient with bladder carcinoma. Elution of antigenic activity from CEA derived from colorectal carcinoma (●) and from UCEA-1 (△). Elution of $\varepsilon_{280}$ from Blue Dextran 2000 (– – –).

minor peak of lower mean molecular size similar to that given when purified (Krupey *et al.*, 1968) CEA (2 mg) derived from colorectal carcinoma was similarly chromatographed.

*Polyacrylamide gel electrophoresis*

The following samples were used: (1) UCEA-1 (20 μg) purified by successive gel filtration on Sepharose 4B and Sephadex G200 followed by Cellogel block electrophoresis (Nery *et al.*, 1974); (2) CEA (20 μg) derived from colorectal carcinoma and obtained as a Sephadex G200 fraction (Krupey *et al.*, 1968) and (3) UCEA-3 (40 μg) obtained after dialysis and freeze drying of the Sepharose 4B fraction A (Fig. 1) of urine 5 (Table). Duplicate samples were subjected to electrophoresis in 15% (w/v) polyacrylamide gels in borate buffer at 3 mA/gel for 7 h in the absence (gels 1–3 respectively) and presence (gels 4–6 respectively) of 5 mol/l urea. The gels were stained for glycoproteins by the periodate-Schiff procedure of Zacharius *et al.* (1969) and for proteins with Coomassie Brilliant Blue. The results (Fig. 3) showed apparently identical protein and glycoprotein bands for UCEA-1 (gel 1) and CEA (gel 2) having an electrophoretic mobility of 0·6–0·7 mm/h per mA, unchanged in the presence of urea (gels 4 and 5 respectively). By contrast, UCEA-3 penetrated the gel poorly in the absence of urea (gel 3) but released, in its presence (gel 6), a diffuse glycoprotein band having a mean electrophoretic mobility similar to that of the other 2 antigens.

*Immunoelectrophoresis on Cellogel strips*

Samples of CEA (30 μg) and of Sepharose 4B sub-fractions A and C (Fig. 1) of urine 5, Table (7 mg and 5 mg respectively), were treated with borate buffered urea (100 μl for the last 2; 120 μl for the first). All but the second gave clear solutions; the second was shaken gently at room temperature overnight and centrifuged for 15 min in a bench centrifuge to give a clear supernatant solution. Triplicate samples (40 μl) of each solution were applied on strips (2·5 cm × 14 cm) of Cellogel and subjected to electrophoresis in borate buffer at 2 mA/cm strip width for 18 min as described by Nery *et al.* (1974). One electrophoretogram was subjected to immunodiffusion against monospecific goat anti-CEA antiserum in a moist chamber at room temperature for 60 h, washed by agitation at room temperature in several changes of barbitone buffer for 16 h and stained with nigrosine. An apparently identical precipitin arc was given by all the samples; UCEA-3 (Sepharose 4B sub-fraction A) also give a slower, less well defined precipitin line (Fig. 4). The other electrophoretograms, stained for glycoprotein by the periodate-Schiff procedure of Bodman (1960), and for

FIG. 3.—Polyacrylamide gel electrophoresis: Electrophoresis was performed in borate buffer in 15% (w/v) gels at 3 mA/gel for 7 h in the absence (gels 1–3) and presence (gels 4–6) of urea. Gels 1 and 4, UCEA-1 (purified Sepharose 4B sub-fraction C, Fig. 1) and gels 3 and 6, UCEA-3 (Sepharose 4B sub-fraction A, Fig. 1) from the urine of a patient with bladder carcinoma; gels 2 and 5, CEA derived from colorectal carcinoma.

FIG. 4.—Immunoelectrophoresis on Cellogel strips: Lanes 1 and 3 contained the dialysed freeze dried products obtained from the Sepharose 4B sub-fractions A and C, respectively, of urine 5 (Table) and lane 2 contained CEA derived from colorectal carcinoma. Immunodiffusion was against undiluted monospecific goat anti-CEA antiserum.

proteins with Coomassie Brilliant Blue, showed the presence of a major band, stained with both reagents, which was coincident with the centre of the common precipitin arc and had a mean electrophoretic mobility of 50–52 mm/h per mA. Other unidentified bands were also revealed for the urinary fractions.

*Identification by immunodiffusion of some components of the UCEA-3 complex*

Petri dishes were coated to a depth of 2 mm with a 1·5% (w/v) solution of agarose in barbitone buffered saline. The peripheral wells (Fig. 5) contained: (1) UCEA-1 (2 mg) obtained from the urine of a patient with bladder carcinoma and purified finally by Cellogel block electrophoresis as described by Nery *et al.* (1974); (2) undiluted monospecific goat anti-CEA antiserum (30 $\mu$l) (Darcy *et al.*, 1973); (3) CEA (20 $\mu$g) derived from colorectal carcinoma and purified finally on Sephadex G200 as described by Krupey *et al.* (1968); and 4–10, undiluted goat anti-human serum or goat anti-human serum components (20 $\mu$l) as follows: (4) anti-IgG ($\gamma$-chain monospecific); (5) anti-whole human serum; (6) anti-IgA ($\alpha$-chain monospecific); (7) anti-IgD ($\delta$-chain monospecific); (8) anti-IgM ($\mu$-chain monospecific); (9) anti-albumin and (10) anti-haptoglobin. The centre wells (Fig. 5a) contained UCEA-3 (4 mg), obtained after dialysis and freeze-drying of combined Sepharose 4B fraction A (Fig. 1) of urine 5 (Table); the centre wells (Fig. 5b) contained the corresponding dialysed and freeze dried fraction of the combined urines (urine 6, Table) of healthy male subjects. Samples of the antigens were transferred to the appropriate wells as solutions in barbitone buffered saline

FIG. 5.—Identification by immunodiffusion of some components of Sepharose 4B sub-fraction A (UCEA-3): Wells in agarose gels contained: (1) UCEA-1; (2) undiluted monospecific goat anti-CEA antiserum; (3) CEA derived from colorectal carcinoma, and (4–10) undiluted goat anti-whole human serum or undiluted goat anti-human serum components, as follows: (4) anti-IgG ($\gamma$-chain monospecific); (5) anti-whole; (6) anti-IgA ($\alpha$-chain monospecific); (7) anti-IgD ($\delta$-chain monospecific); (8) anti-IgM ($\mu$-chain monospecific); (9) anti-albumin and 10, anti-haptoglobin. The centre wells contained Sepharose 4B sub-fraction A of urine (urine 5, Table) from a patient with bladder carcinoma (Fig. 5a) or of urine (urine 6) from healthy male subjects (Fig. 5b).

(50–100 $\mu$l). After immunodiffusion in a moist chamber at room temperature for 72 h, the immunograms were washed in several changes of the same buffer by periodical agitation at room temperature for 24 h and photographed. The results (Fig. 5 and Table) show the presence of components of the UCEA-3 complex giving reactions of antigenic identity with CEA derived from colorectal carcinoma and others having antigenic specificities characteristic of albumin, haptoglobin and the heavy chains of IgG and IgA. The other UCEA-3 complexes (Fractions A, Fig. 1, of urines 1–3, Table) also showed the presence of these components in varying amounts; in addition, those from urines 1 and 3 showed the presence of a component having antigenic specificity characteristic of the heavy chain of IgM. Fraction A (Fig. 1) of the perchloric acid soluble components of urine 5 (Table) showed the presence of substances bearing the IgG and IgM, but not the other specificities. The corresponding complex from fraction A (Fig. 1) of urine (urine 6, Table) from healthy male subjects showed components having the albumin and IgG but not the haptoglobin, IgA or IgM specificities detectable under the conditions described (Fig. 5b). The results are summarized in the Table.

### DISCUSSION

The present study describes 3 apparently characteristic properties of urinary substances which cross-react during radioimmunoassay with CEA derived from colorectal carcinoma. These properties are heterogeneity in molecular size, a pronounced tendency to occur in large multi-component aggregates and low specific activity, *i.e.* weight equivalent antigenic activity per unit weight of the substance.

Heterogeneity in molecular size was evident from the wide distribution of antigenic activity in Sepharose 4B fractions of all samples of urines investigated

(Fig. 1 and Table). Conceivably, this heterogeneity might have resulted from interactions of a single CEA-like glycoprotein (Component C, Fig. 1) having a mean molecular size approximately similar to that ($2 \times 10^5$; Krupey *et al.*, 1968) of CEA derived from colorectal carcinoma; the larger (A and B, Fig. 1) or smaller (D) components would then have arisen from aggregation or hydrolysis by urinary hydrolases, respectively, of component C. This appears unlikely for several reasons: (a) A Sephadex G200 fraction of component C (UCEA-1) derived from the urine of a patient with bladder carcinoma and a similar fraction of CEA derived from colorectal carcinoma were not appreciably degraded after incubation at 37°C for 16 h of solutions of the antigens in urine from healthy subjects or from patients with bladder carcinoma (James, Neville and Nery, unpublished). (b) The specific activities ((*b*), Table) of components S and A–D (Table) of the various urines showed wide variations and no consistent pattern emerged. (c) The highest specific activity (0·51%) occurred in component C of a perchloric acid soluble fraction (urine 5a) of urine from a patient with bladder carcinoma. Further purification of this component by successive gel filtration on Sephadex G200 and Cellogel block electrophoresis gave a final product having a specific activity of 3% (Nery *et al.*, 1974). By contrast, the corresponding Sepharose 4B and Sephadex G200 fractions of perchloric acid extracts of colorectal carcinoma contained specific activities of 40–80% and 60–100% respectively (Krupey *et al.*, 1968; Coligan *et al.*, 1973; Turberville *et al.*, 1973).

Some urinary CEA-like substances occurred in large multi-component aggregates. The aggregations were probably neither due to contamination with cervical secretions nor induced by experimental procedures involving freezing and thawing of the urines, followed by freeze drying of the various dialysed fractions. Urine collected by catheterization of the bladder of a patient with bladder carcinoma showed similar distributions of antigenic activity during Sepharose 4B chromatography whether such procedures were employed or eliminated. Sepharose 4B fractions (Fig. 1 and Table), showing mean molecular weights of over 20 million (Fraction A) and intermediate (Fraction B) between this and $2 \times 10^5$ (*i.e.* the mean molecular weight of fractions C and of CEA derived from colorectal carcinoma) released components which were qualitatively indistinguishable from CEA derived from colorectal carcinoma in several respects: (a) Such components were released (i) during perchloric acid extraction of urine 5 (compare curves 1 and 2 of Fig. 5, obtained before and after extraction, respectively) and during gel filtration on Sepharose 2B (Fig. 2) of the perchloric acid soluble Sepharose 4B fraction A of the same urine, indicating similarity in molecular size and (ii) during electrophoresis in polyacrylamide gels (Fig. 3) and immunoelectrophoresis on Cellogel (Fig. 4), indicating similarity in electrophoretic mobility and antigenic properties. (b) Precipitin lines of antigenic identity formed during immunodiffusion in agarose against monospecific anti-CEA antiserum (Fig. 5) indicated the presence of common or cross-reacting antigenic determinants. (c) Reactions characteristic of glycoproteins, *i.e.* formation of complexes with borate ions and staining with periodate-Schiff reagent and with Coomassie Brilliant Blue, were given by both substances (Fig. 3 and 4). (d) Treatment of Sepharose 4B fraction A (Fig. 1) of urine 5 (Table) with 5 mol/l urea caused disaggregation releasing at least 2 components bearing antigenic similarities to CEA derived from colorectal carcinoma (Fig. 4). Urinary components showing reactions of partial antigenic identity with CEA have also been detected (Darcy, personal communication, and Nery *et al.*, 1974). Sepharose 4B fractions A and B also contained substances (in variable amounts and occurrence) showing antigenic specicities of albumin, haptoglobin and the heavy chains of immunoglobulins G, A

and M (Fig. 5). The presence of the heavy chain IgM specificity (Nery and James, unpublished) in preparations of UCEA-1 (mean mol. wt $2 \times 10^5$) may thus be due to the separated $\mu$-chains and not to the intact IgM molecule (mol. wt. $9 \times 10^5$). Further, perchloric acid solubilized only part (79%) of the CEA-like activity giving a macromolecular aggregate (Fraction A, Fig. 1 of urine 5, Table) containing immunoglobulin specificities (Table); it is thus possible that some of the immunoglobulins or their component heavy chains are attached to CEA-like components of varying carbohydrate composition by covalent, *e.g.* disulphide, bonds not disrupted by acid treatment and resulting in precipitation of the CEA-like components of low carbohydrate composition.

The low specific activity (columns (*b*) of Sepharose 4B fractions S and A–D, Table) was not significantly different in urine (urine 6) of healthy male subjects and in urine (urines 1–5) of cancer patients. The higher antigenic activity found in the latter urines (Hall *et al.*, 1972) might thus have resulted in part from the higher concentration (approximately 1 g/l and 0·1 g/l of urine from the cancer patients and normal subjects respectively) of urinary components containing CEA-like activity and having an approximate molecular weight greater than $3 \times 10^4$. Since an approximately ten-fold difference in urinary titre between the cancer patients and healthy subjects has not been observed (Table, column C), other determining factors might have been the multiplicity, competitive reactivity and accessibility of CEA-like antigenic determinant groups. Such groups might have been hidden in aggregates by direct combination, *e.g.* with the immunoglobulin components or by steric factors; they might have been exposed during partial disaggregations induced during gel filtration on Sepharose 4B, resulting in recoveries of antigenic activities generally greater than 100% and exceeding 1500% in one case (urine 3, Table). The recovery of less than 100% observed in urine 4 (Table) might have been due to the presence of dialysable CEA-like components in some of the aggregates. The presence of such components in urines of patients with bladder carcinoma has been reported (Nery *et al.*, 1974). Some of these components might have been freed from aggregation and lost during the extensive dialysis of the Sepharose 4B fractions. Comparison of these fractions of urine 4 shows the highest proportion of antigenic activity in Fraction B before (curve 4 of Fig. 1), but the lowest after (0·9 $\mu$g or 3·6% of the total eluted activity) the fractions were dialysed. The dialysed fractions also showed the highest relative proportions of CEA-like components of molecular sizes greater ($A_c + B_c/C_c$, Table) than that of fraction C, with the bulk (80%) of the non-dialysable activity now occurring in the fraction (Fraction A) of greatest molecular size.

This and other studies (Martin and Martin, 1970; Freed and Taylor, 1972; Laurence *et al.*, 1972; Reynoso *et al.*, 1972; Nery *et al.*, 1973) showing the presence of substances giving positive values during radioimmunoassays for CEA in both normal and diseased states indicate that such substances may represent a family of antigenically related molecules. These substances are generally secreted in greater amounts during injury and disease and may differ from one another by, as yet undetermined, structural modifications of normal tissue components as a result of impaired cellular metabolism.

The authors thank Dr C. W. Todd (City of Hope National Medical Center, Duarte, California) for generous gifts of the standard CEA and horse anti-goat IgG antiserum, and Dr D. Darcy and Dr C. Turberville (both of this Institute) for generous gifts of goat monospecific anti-CEA antiserum and CEA respectively. This investigation was supported by the Medical Research Council (Grants 970/656/B and 971/817/B).

### REFERENCES

BODMAN, J. (1960) Agar Gel, Starch, Block, Starch Gel and Sponge Rubber Electrophoresis. In *Chromatographic and Electrophoretic Techniques. Vol.* 2. *Zone Electrophoresis*. Ed. I. Smith. London: Heinemann. p. 137.

COLIGAN, J. E., HENKART, P. A., TODD, C. W. & TERRY, W. D. (1973) Heterogeneity of the Carcinoembryonic Antigen. *Immunochemistry*, **10**, 591.

DARCY, D. A., TURBERVILLE, C. & JAMES, R. (1973) Immunological Study of Carcinoembryonic Antigen (CEA) and a Related Glycoprotein. *Br. J. Cancer*, **28**, 147.

DAVIS, B. J. (1964) Disc Electrophoresis-II. Method and Application to Human Serum Proteins. *Ann. N.Y. Acad. Sci.*, **121**, 404.

EGAN, M. L., LAUTENSCHLEGER, J. T., COLIGAN, J. E. & TODD, C. W. (1972) Radio-immune Assay of Carcinoembryonic Antigen. *Immunochemistry*, **9**, 289.

FREED, D. L. & TAYLOR, G. (1972) Carcinoembryonic Antigen in Faeces. *Br. med. J.*, i, 85.

GOLD, P. & FREEDMAN, S. O. (1965) Demonstration of Tumour-specific Antigens in Human Colonic Carcinoma by Immunological Tolerance and Absorption Technique. *J. exp. Med.*, **121**, 439.

HALL, R. R., LAURENCE, D. J. R., DARCY, D., STEVENS, U., JAMES, R., ROBERTS, S. & NEVILLE, A. M. (1972) Carcinoembryonic Antigen (CEA) in the Urine of Patients with Urothelial Carcinoma. *Br. med. J.*, iii, 609.

KRUPEY, J., GOLD, P. & FREEDMAN, S. O. (1968) Physicochemical Studies of the Carcinoembryonic Antigen of the Human Digestive System. *J. exp. Med.*, **128**, 387.

LAURENCE, D. J. R., STEVENS, U., BETTELHEIM, R., DARCY, D., LEESE, C., TURBERVILLE, C., ALEXANDER, P., JOHNS, E. W. & NEVILLE, A. M. (1972) Evaluation of the Role of Plasma Carcinoembryonic Antigen (CEA) in the Diagnosis of Gastrointestinal, Mammary and Bronchial Carcinoma. *Br. med. J.*, iii, 605.

MARTIN, F. & MARTIN, M. S. (1970) Demonstration of the Antigens Related to Colonic Cancer in the Human Digestive System. *Int. J. Cancer*, **6**, 352.

NERY, R., BARSOUM, A. L. & BULLMAN, H. (1973) Carcinoembryonic Antigens of Erythrocyte Membranes. *Nature, New Biol.*, **246**, 44.

NERY, R., BARSOUM, A. L., BULLMAN, H. & NEVILLE, A. M. (1974) Carcinoembryonic Antigen-like Substances of Human Urothelial Carcinomas. Isolation of Components from Pathological Urine and Comparison with Colorectal Carcinoma Antigens. *Biochem. J.*, In the press.

REYNOSO, G., CHU, T. M., HOLYOKE, D., COHEN, D., NAMOTO, T., WANG, J. J., CHUANG, J., GUINAN, P. & MURPHY, G. D. (1972) Carcinoembryonic Antigen in Patients with Different Cancers. *J. Am. med. Ass.*, **220**, 361.

TURBERVILLE, C., PELLY, J., JOHNS, E. W., DARCY, D. & LAURENCE, D. J. R. (1973) Purification and Characterisation of Carcinoembryonic Antigen from Human Colonic Carcinomas. *Biochem. Soc. Trans. 536th Meeting*, **1**, 611.

ZACHARIUS, R. M., ZELL, T. E., MORRISON, J. H. F. & WOODLOCK, J. J. (1969) Glycoprotein Staining Following Electrophoresis on Acrylamide Gels. *Analyt. Biochem.*, **30**, 148.


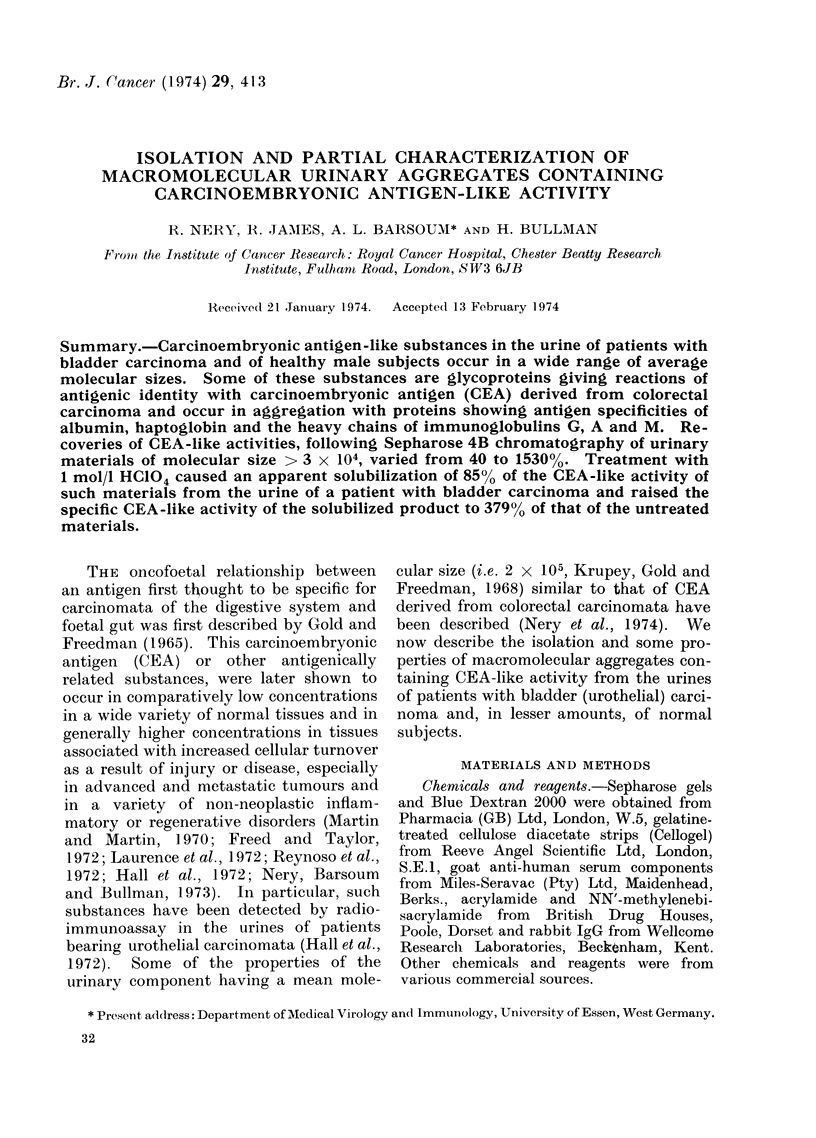

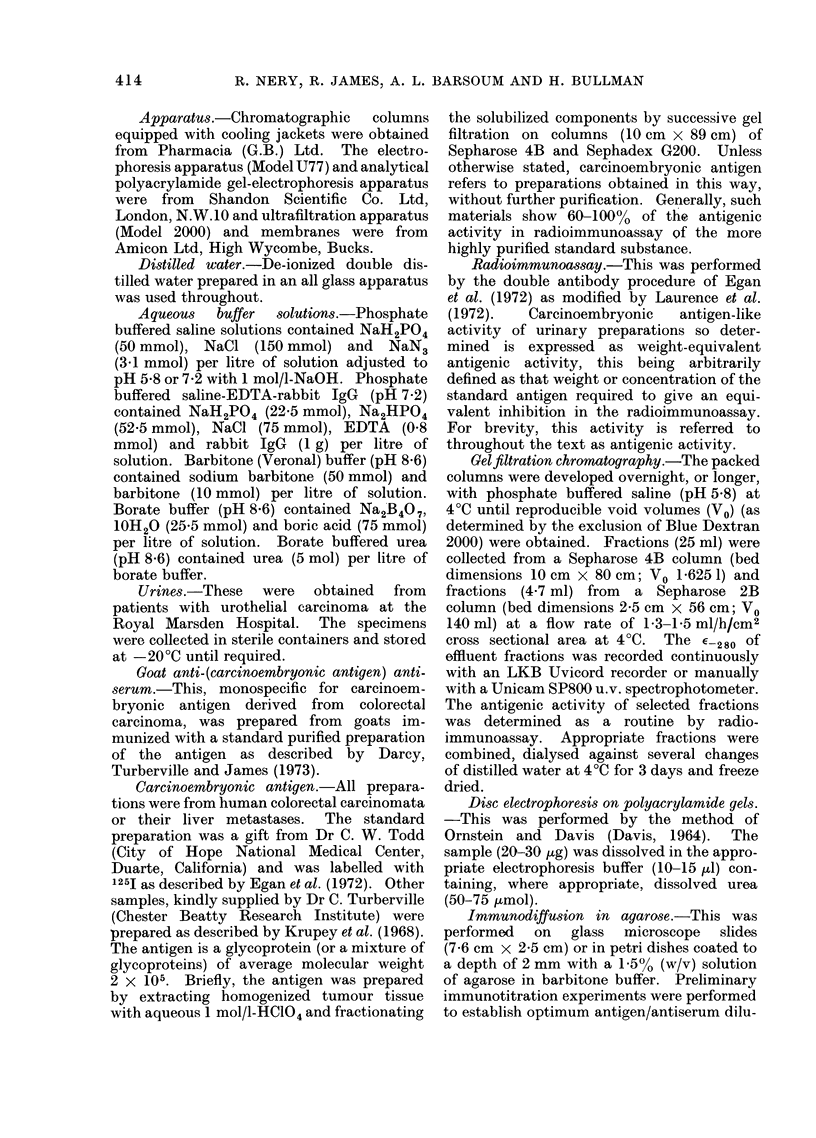

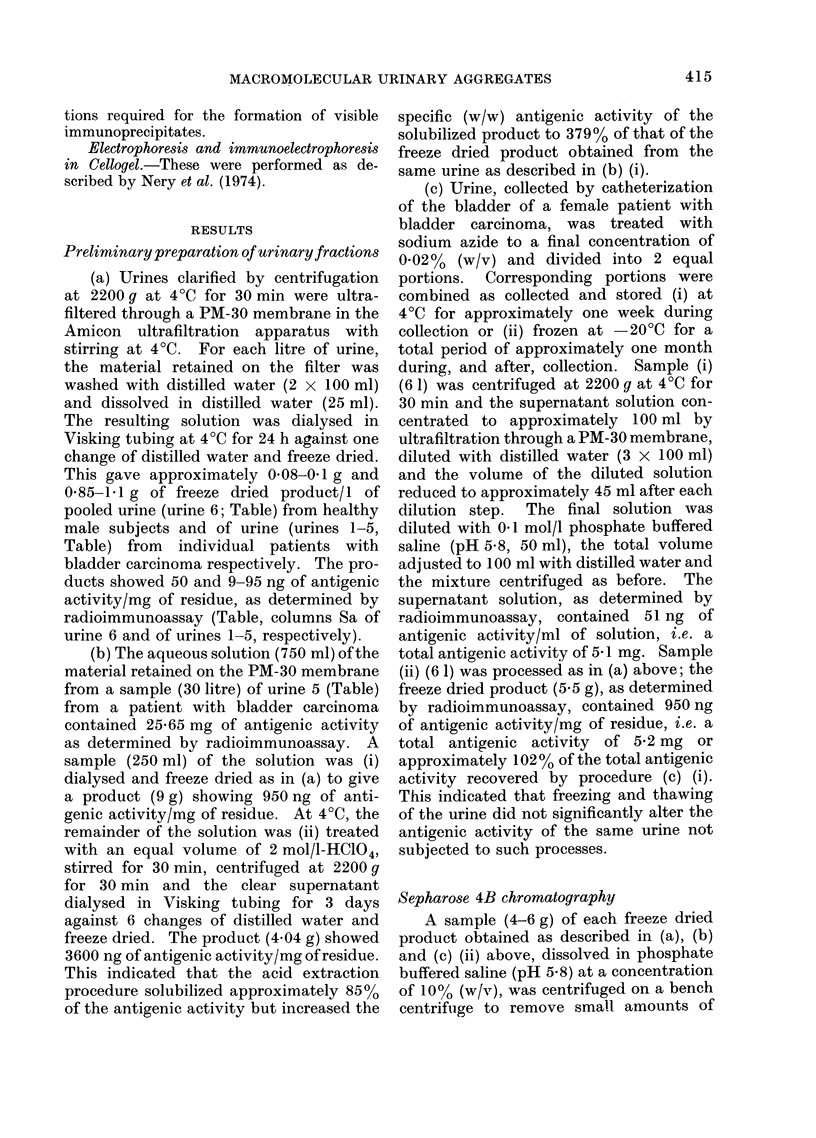

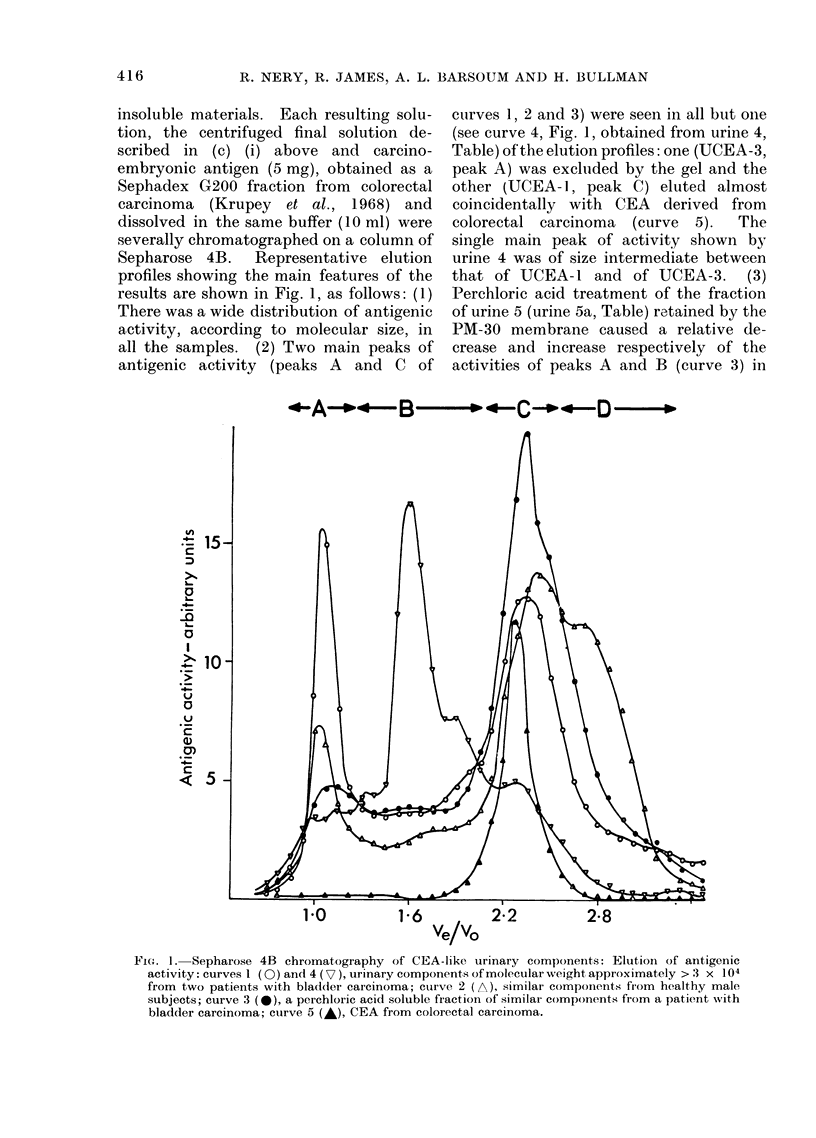

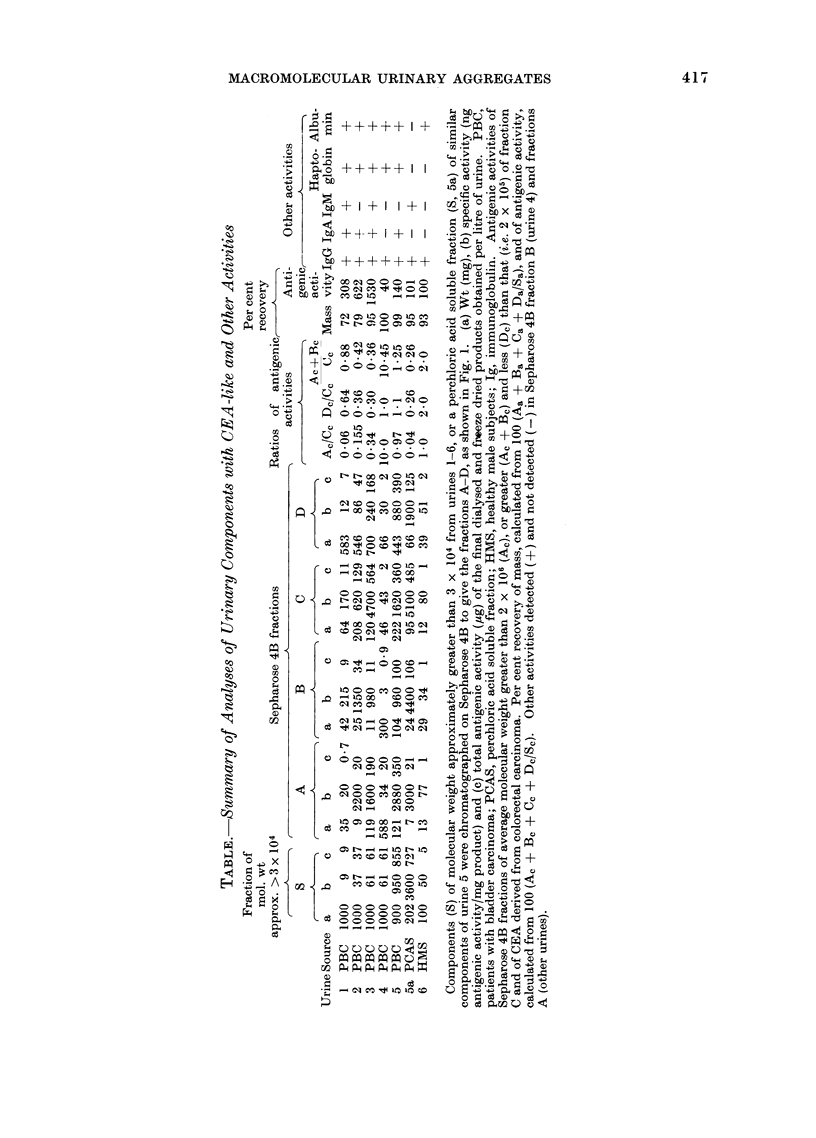

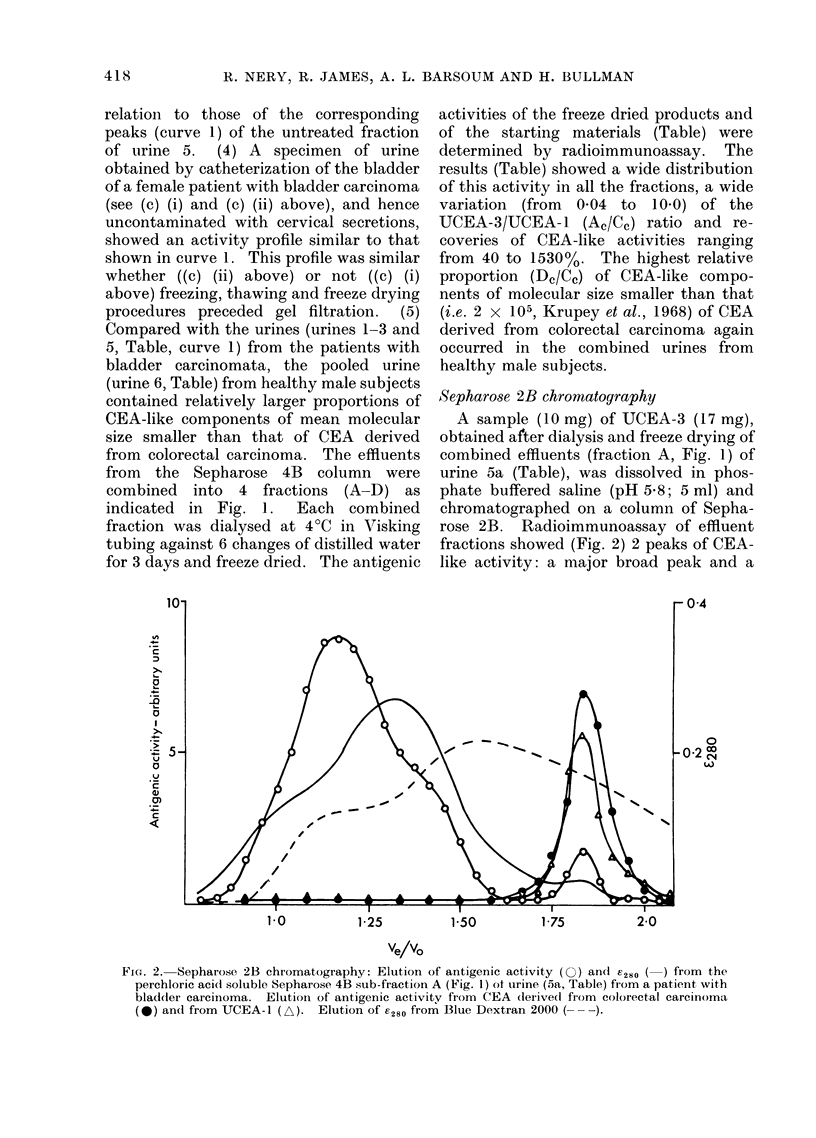

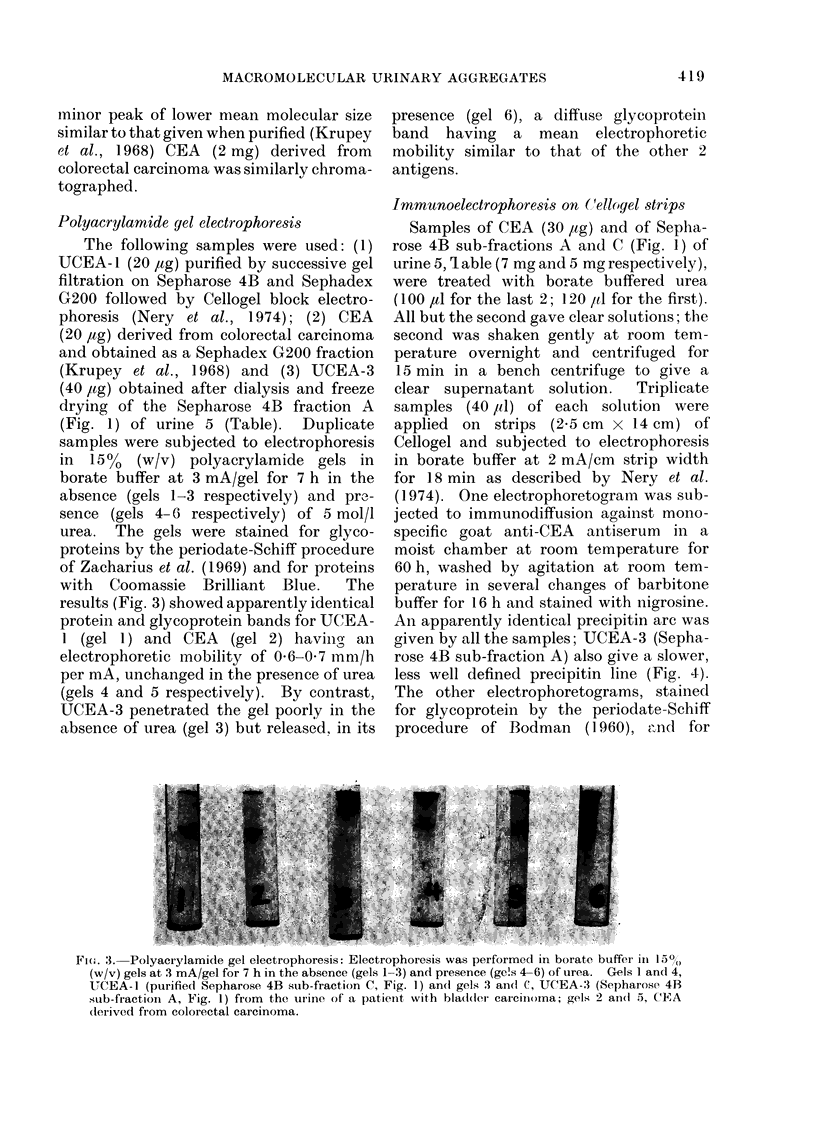

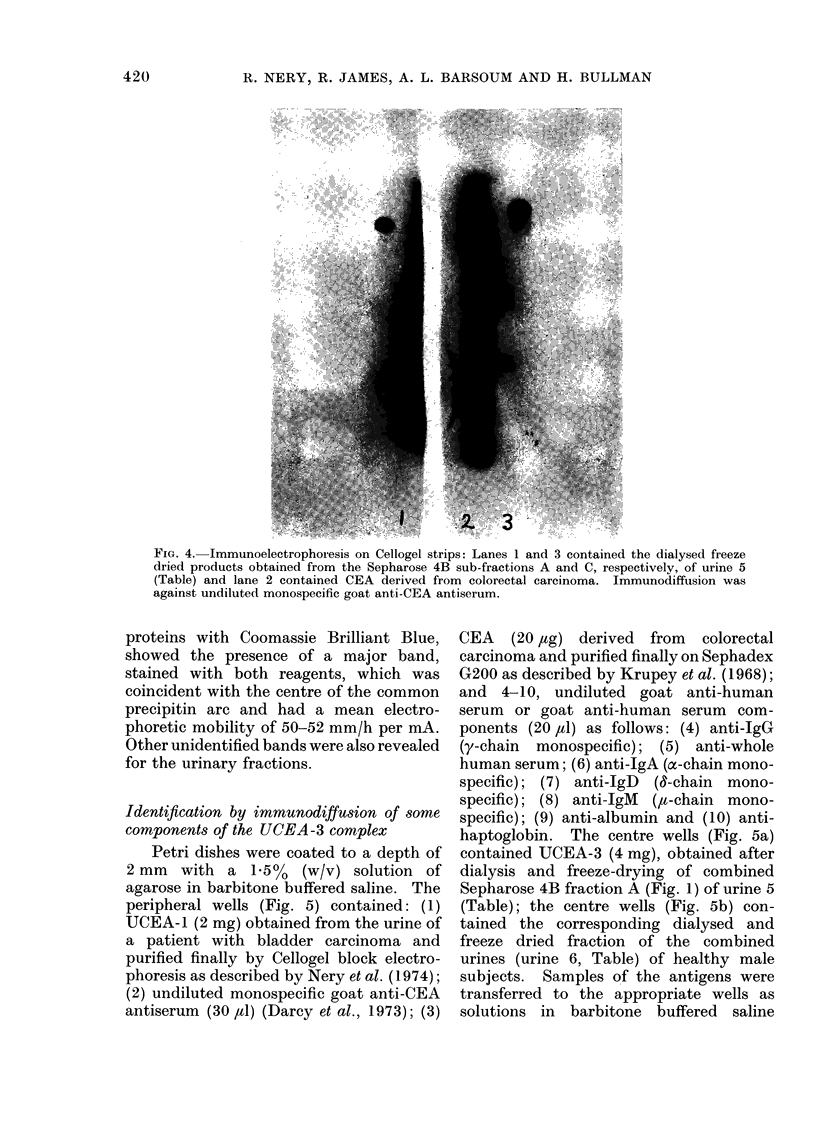

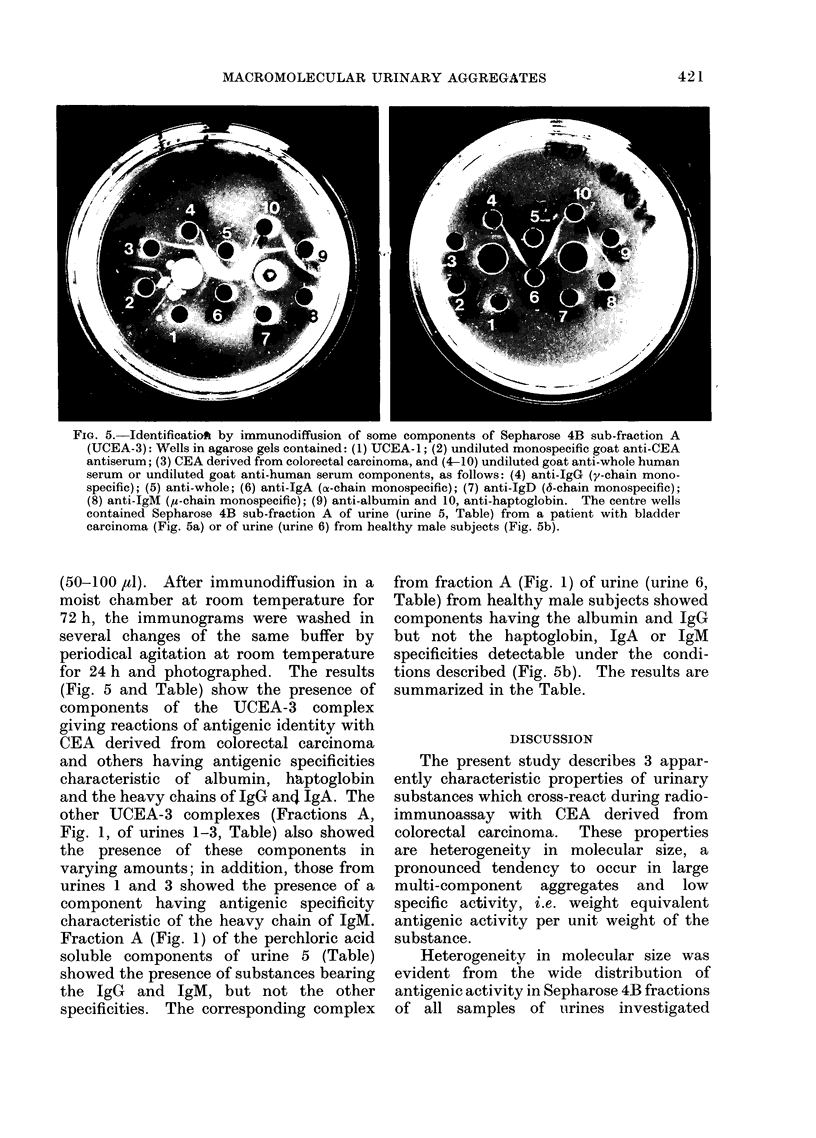

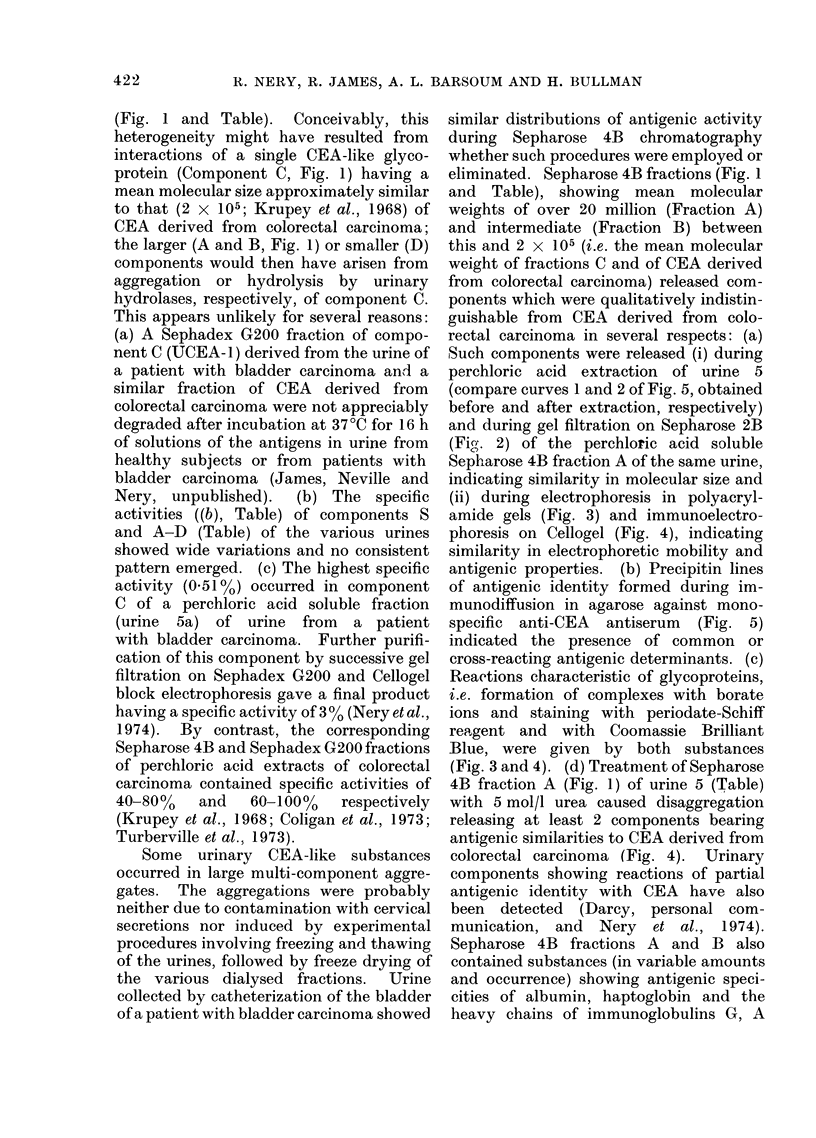

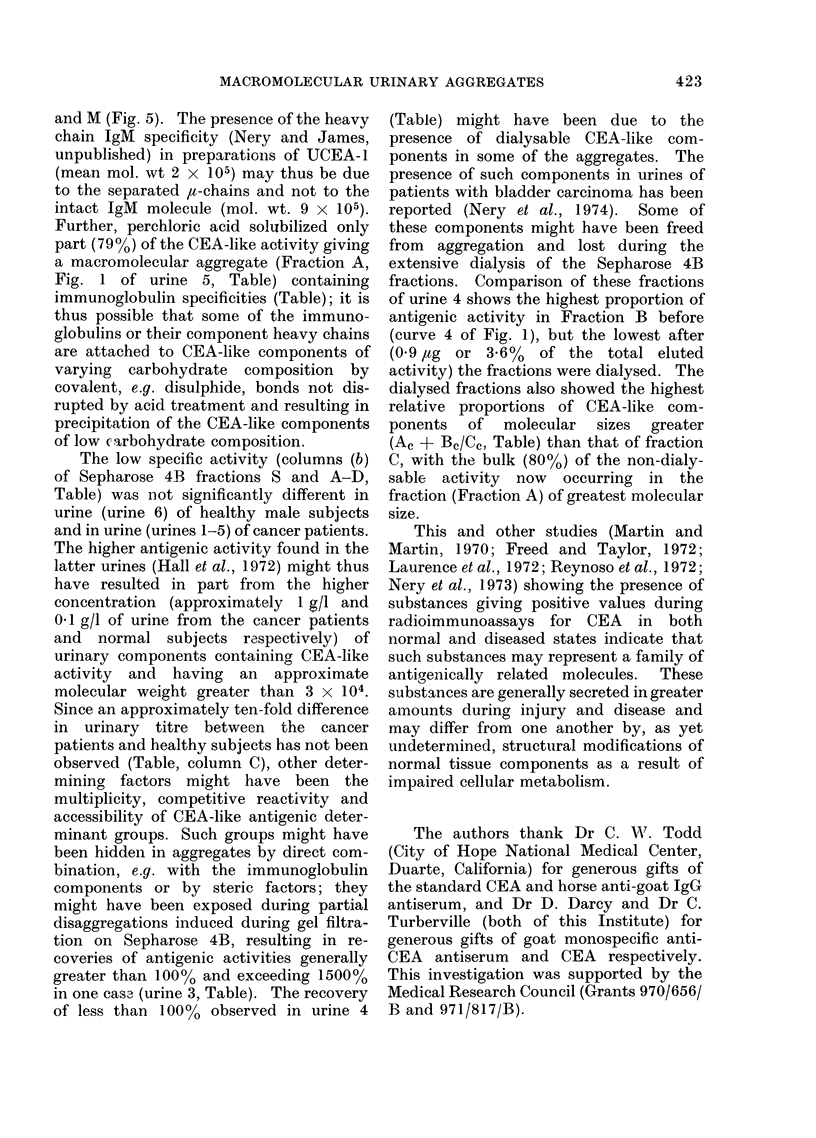

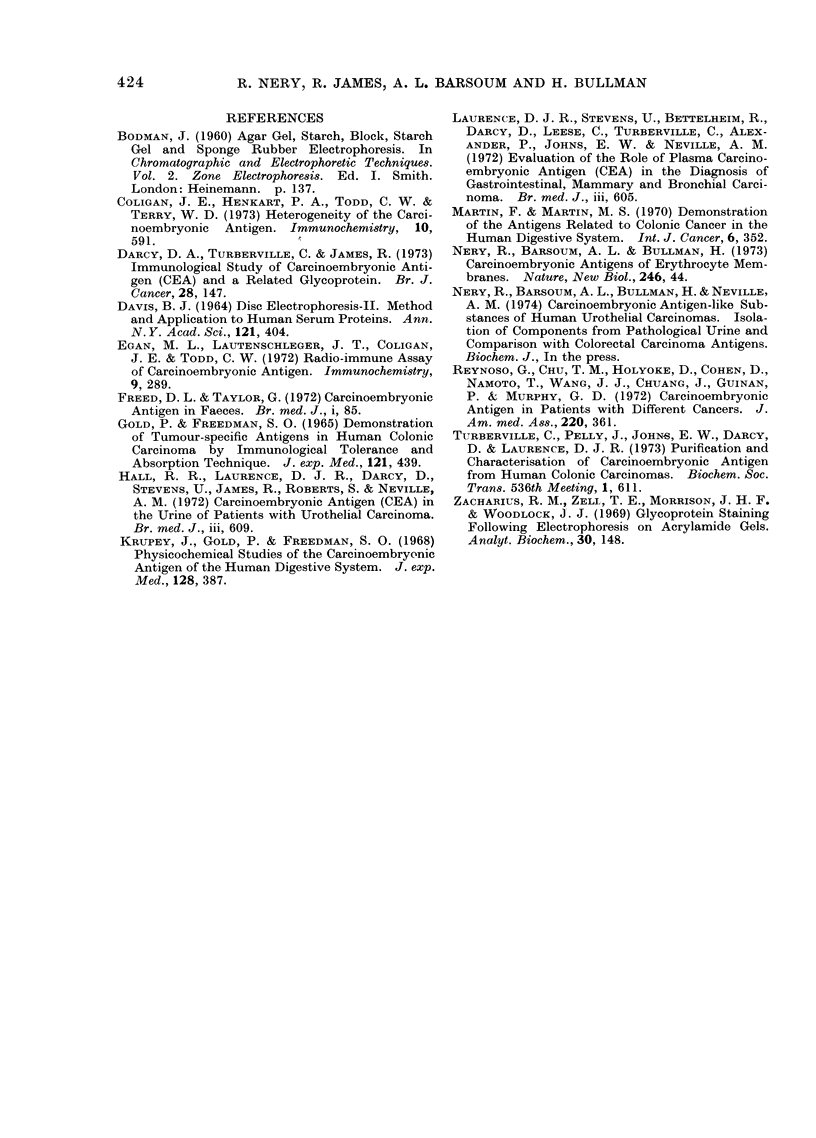


## References

[OCR_01011] DAVIS B. J. (1964). DISC ELECTROPHORESIS. II. METHOD AND APPLICATION TO HUMAN SERUM PROTEINS.. Ann N Y Acad Sci.

[OCR_01005] Darcy D. A., Turberville C., James R. (1973). Immunological study of carcinoembryonic antigen (CEA) and a related glycoprotein.. Br J Cancer.

[OCR_01016] Egan M. L., Lautenschleger J. T., Coligan J. E., Todd C. W. (1972). Radioimmune assay of carcinoembryonic antigen.. Immunochemistry.

[OCR_01022] Freed D. L., Taylor G. (1972). Carcinoembryonic antigen in faeces.. Br Med J.

[OCR_01026] GOLD P., FREEDMAN S. O. (1965). DEMONSTRATION OF TUMOR-SPECIFIC ANTIGENS IN HUMAN COLONIC CARCINOMATA BY IMMUNOLOGICAL TOLERANCE AND ABSORPTION TECHNIQUES.. J Exp Med.

[OCR_01032] Hall R. R., Laurence D. J., Darcy D., Stevens U., James R., Roberts S., Munro Neville A. (1972). Carcinoembryonic antigen in the urine of patients with urothelial carcinoma.. Br Med J.

[OCR_01039] Krupey J., Gold P., Freedman S. O. (1968). Physicochemical studies of the carcinoembryonic antigens of the human digestive system.. J Exp Med.

[OCR_01048] Laurence D. J., Stevens U., Bettelheim R., Darcy D., Leese C., Turberville C., Alexander P., Johns E. W., Neville A. M. (1972). Role of plasma carcinoembryonic antigen in diagnosis of gastrointestinal, mammary, and bronchial carcinoma.. Br Med J.

[OCR_01054] Martin F., Martin M. S. (1970). Demonstration of antigens related to colonic cancer in the human digestive system.. Int J Cancer.

[OCR_01058] Nery R., Bullman H., Barsoum A. L. (1973). Carcinoembryonic antigens of erythrocyte membranes.. Nat New Biol.

[OCR_01071] Reynoso G., Chu T. M., Holyoke D., Cohen E., Nemoto T., Wang J. J., Chuang J., Guinan P., Murphy G. P. (1972). Carcinoembryonic antigen in patients with different cancers.. JAMA.

[OCR_01085] Zacharius R. M., Zell T. E., Morrison J. H., Woodlock J. J. (1969). Glycoprotein staining following electrophoresis on acrylamide gels.. Anal Biochem.

